# Development of Surface Molecularly Imprinted Polymers as Dispersive Solid Phase Extraction Coupled with HPLC Method for the Removal and Detection of Griseofulvin in Surface Water

**DOI:** 10.3390/ijerph17010134

**Published:** 2019-12-24

**Authors:** Kamran Bashir, Zhimin Luo, Guoning Chen, Hua Shu, Xia Cui, Wen Li, Wang Lu, Qiang Fu

**Affiliations:** School of Pharmacy, Xi’an Jiaotong University, Xi’an 710061, China; kamranpharmacist27@gmail.com (K.B.); luozm0905@xjtu.edu.cn (Z.L.); chengn1992@stu.xjtu.edu.cn (G.C.); shuhuas@stu.xjtu.edu.cn (H.S.); cuixia0415@stu.xjtu.edu.cn (X.C.); liwen19940820@stu.xjtu.edu.cn (W.L.); wanglu0914@stu.xjtu.edu.cn (W.L.)

**Keywords:** dispersive solid phase extraction, griseofulvin, HPLC, removal and analysis, pharmaceutical pollutants, surface water

## Abstract

Griseofulvin (GSF) is clinically employed to treat fungal infections in humans and animals. GSF was detected in surface waters as a pharmaceutical pollutant. GSF detection as an anthropogenic pollutant is considered as a possible source of drug resistance and risk factor in ecosystem. To address this concern, a new extraction and enrichment method was developed. GSF-surface molecularly imprinted polymers (GSF-SMIPs) were prepared and applied as solid phase extraction (SPE) sorbent. A dispersive solid phase extraction (DSPE) method was designed and combined with HPLC for the analysis of GSF in surface water samples. The performance of GSF-SMIPs was assessed for its potential to remove GSF from water samples. The factors affecting the removal efficiency such as sample pH and ionic strength were investigated and optimized. The DSPE conditions such as the amount of GSF-SMIPs, the extraction time, the type and volume of desorption solvents were also optimized. The established method is linear over the range of 0.1–100 µg/mL. The limits of detection and quantification were 0.01 and 0.03 µg/mL respectively. Good recoveries (91.6–98.8%) were achieved after DSPE. The intra-day and inter-day relative standard deviations were 0.8 and 4.3% respectively. The SMIPs demonstrated good removal efficiency (91.6%) as compared to powder activated carbon (67.7%). Moreover, the SMIPs can be reused 10 times for water samples. This is an additional advantage over single-use activated carbon and other commercial sorbents. This study provides a specific and sensitive method for the selective extraction and detection of GSF in surface water samples.

## 1. Introduction

The presence of pharmaceutical substances in the environmental waters has become a global hazard [1,2]. The major sources of these contaminations are industries, agriculture and long term drought. The surface water and ground water have been seriously affected by these pollutants. These chemicals enter into the body systems of humans, animals and aquatic strains through the food chain. Their intended or un-intended use and excretion in discharge waters pose potential harmful effects on the public health and aquatic ecosystem [3,4].

Antibiotics, an important group of pharmaceuticals, have been excessively used around the globe. Meanwhile, antibiotic pollution has also posed a major global threat to humans [5]. The easy migration of antibiotics in drinking water causes serious drug resistance which brings the environmental risk in terms of residual antibiotics released into the ecosystem [6]. Griseofulvin (GSF), one of the major constituents of antibiotics, is among the first discovered oral antifungal drugs and extensively used in treating mycotic infection both in animals and humans. GSF appears as white to pale crystalline powder, odorless and tasteless. It is very less soluble in water and soluble in organic solvents. This drug has been used for more than forty years and is a drug of choice for the treatment of *Tinea capitis* [7]. GSF new properties like antiviral and anticancer was studied in the mammalian system and revived its clinical use other than an anti-mycotic drug [8,9,10]. Despite its clinical applications, multiple severe side effects have also been associated with its clinical use [11]. A variety of acute and chronic toxic effects such as teratogenicity, liver and thyroid cancers, embryo toxicity and abnormal germ cell maturation were also reported [12]. Phytotoxic effect on plants, inhibition, delay and abnormalities induced by GSF in stent or have been reported. Hashimoto in 1969 reported GSF as less toxic to fish and aquatic organisms [13,14,15]. According to literature survey, GSF has been detected in Miño River, Spain from a rural site at a maximum concentration of 37.6 ng/L [16]. Another study reported its presence in Romanian surface water samples [17]. GSF was also reported as a surface water and waste waters pharmaceutical pollutant [18,19,20]. GSF is produced as a byproduct of *Penicillium griseofulvum* and could be a possible source of continuous excretion in aquatic environment [21]. Additionally, the continuous environmental exposure from water sources may cause unnecessary adverse effects in aquatic environment and can harm animals and humans by food chain. Therefore, its separation and detection in environmental water is crucial. The extraction and estimation of GSF have been reported in food, molds, aquatic strains, [22] but the detection of GSF in Chinese surface water samples was not studied previously.

Considering the threats of GSF in surface water, the general waste water treatment procedures have been handled, such as physical adsorption, oxidation or bioremediation. However, GSF, as a micro-pollutant (whose concentration is extremely low), is hard to be completely removed. Therefore, its detection and removal is still a challenge. Typically, GSF is analyzed or detected by high performance liquid chromatography, gas chromatography and liquid chromatography-mass spectrometry, etc. [23]. Whereas, a reliable sample pretreatment is required before using these instruments. Generally, solid phase extraction (SPE) is used in sample pretreatment. However, the traditional SPE sorbents, such as C18, C8, Florisil, Silica and Charcoal have no selectivity for the targeted compounds [24]. In recent years, molecularly imprinted polymers (MIPs) have been considered as attractive adsorbent to produce artificial receptors which are complementary in size, shape, and functional groups to the template molecules [25]. The MIP prepared by surface imprinting approach has more recognition sites at the surface of material which encourage a quick mass exchange, fast recognition and more recoveries. The surface molecularly imprinted polymers (SMIPs) show great strength at different pH values, extraordinary mechanical quality at different temperatures, and robustness in various solvents [26]. SPE advanced subdivisions such as dispersive SPE (DSPE), stir-bar sportive extraction, solid phase micro extraction, and single drop micro extraction, are considered as highly selective, less expensive and more environment friendly as compared to conventional extraction methods [27,28,29]. The dispersive extraction method is designed to increase the surface area between sample solution and sorbents, decrease extraction time and decrease the amount of sorbent by means of dispersion [30,31,32]. The procedure requires the utilization of exceptionally proficient extraction media so as to keep up or increase the pre-concentration of analytes in samples and by using just few milligrams or microliters of extraction media.

The aim of this study was to prepare and apply SMIPs as DSPE sorbents for specific and selective separation and enrichment of GSF from complex matrix, and to establish the SMIP-DSPE coupled to HPLC method for the sensitive and selective analysis of GSF in surface water samples. We also studied the factors affecting removal efficiency and optimized the DSPE conditions. This is the first report for the selective removal and extraction of GSF from surface water samples.

## 2. Methods

### 2.1. Chemical and Instruments

GSF, voriconazole and miconazole were purchased from Aladdin Co. (Beijing, China). Ultra-pure water was purified with Molement 1805 b (Shanghai, China). Methanol and acetonitrile were of HPLC grade. All other chemicals used in this study were supplied from local suppliers and of analytical grade.

The HPLC analysis was performed with a Shimadzu LC-2010AHT series HPLC (Kyoto, Japan) and a Lab-Solution workstation. The shaking was performed by a SHA-C Vapour-Bathing Constant Temperature Vibrator (Changzhou, China). For chromatographic separation, a Promasil C_18_ column (250 mm × 4.6 mm, 5 μm) was used. The mobile phase was methanol-water (70:30, *v*/*v*%) and a flow rate of 1.0 mL/min was used. The detection wavelength was 291 nm, and the column oven temperature was set at 30 °C.

### 2.2. Drug Standards, Spiked Samples and Surface Water Collection

A stock solution (1000 µg/mL) of GSF was prepared in methanol. Further the working standard solutions of 0.1, 1, 10, 25, 50, 75, 100, 200, 300, 400, 500 µg/mL prepared by serially diluted with methanol-water (70:30, *v*/*v*).

The surface water collected from Qing Ling Lake in Xi’an, Shaanxi province was filtered through a membrane of 0.45 µm pore size. The surface water samples and deionized water samples were spiked with GSF working standard solution concentrations (0.1–500 µg/mL) for further analysis.

### 2.3. Preparation and Characterization

The GSF-SMIPs were prepared by using modified silica gel particles as a support material and by using non-covalent surface imprinting method. GSF (0.5 mM) was added in toluene (20 mL), methacrylic acid (2 mM) was used as functional monomer, ethylene glycol dimethacrylate (10 mM) as cross linker, 2,2′-azobisisobutyronitrile (16.4 mg) as an initiator and toluene was used as a solvent. After polymerization the polymers were collected, washed and template was removed by using methanol-acetic acid (4:1). The surface non imprinted polymers (SNIPs) were prepared without template drug by the same method. Detail preparation and characterization were performed and discussed in our previous work [33].

### 2.4. Removal of GSF in Surface Water

#### 2.4.1. Removal Efficiency in Surface Water and Deionized Water Samples

In this experiment, 10 mg of SMIPs were added in 10 mL flask containing 10.0 mL of spiked surface water and deionized water of different concentrations (10–500 µg/mL). After being shaken for 30 min at room temperature, the samples were centrifuged and filtered and were analyzed by HPLC. The adsorption quantities Q (µg/mg) of SMIPs and SNIPs were estimated by the following equation.

Q = (C_o_ − C_f_)*V/m (1)

‘C_o_’ is the initial concentration and ‘C_f_’ (μg/mL) is the concentration after adsorption, ‘V’ is volume of drug solution and ‘m’ (mg) is the amount of polymer used. The adsorbed amount was the measure of removal efficiency. All the experiments were performed in triplicate.

#### 2.4.2. Effect of pH

To examine the effect of pH on removal efficiency, we adjusted the pH of drug solution (100 µg/mL) from 1–10 by adding HCl or NaOH. Then 10.0 mL drug solutions of different pH were added in separate flasks. In each flask 50 mg of SMIPs and 50 mg SNIPs were dispersed. Each flask was shaken for 30 min at room temperature. The samples were centrifuged and filtered and analyzed by HPLC. The final removal(%) was determined.

#### 2.4.3. Effect of Ionic Strength

In this experiment, NaCl salt (0, 1, 2, 3, 4, 5 and 6%) was added in drug solution (100 µg/mL). After this, 50 mg of SMIPs and SNIPs were added in the different reaction flasks. The flasks were shaken for 30 min at room temperature. After this the samples were centrifuged and filtered. The removal (%) was calculated.

#### 2.4.4. Removal Efficiency of SMIPs, SNIPs and Activated Carbon

For this experiment, powder activated carbon PAC was treated with 0.1 mol/L of hydrochloric acid in order to remove ions [34] After this, the pH of the PAC was neutralized by washing with deionized water. The PAC was dried at 100 °C. After this, 50 mg of SMIPs, SNIPs and PAC were added in different reaction flasks and then 10 mL of drug solution (100 µg/mL) were added in respective flasks. The flasks were shaken for 1 h, at room temperature; then the samples were separated, centrifuged and filtered. The removal (%) was measured.

### 2.5. DSPE Procedure for the Extraction of GSF

In this experiment we utilized GSF-SMIPs as a selective sorbent and a DSPE method was combined with HPLC for the analysis of GSF in water samples.

**Figure 1:**
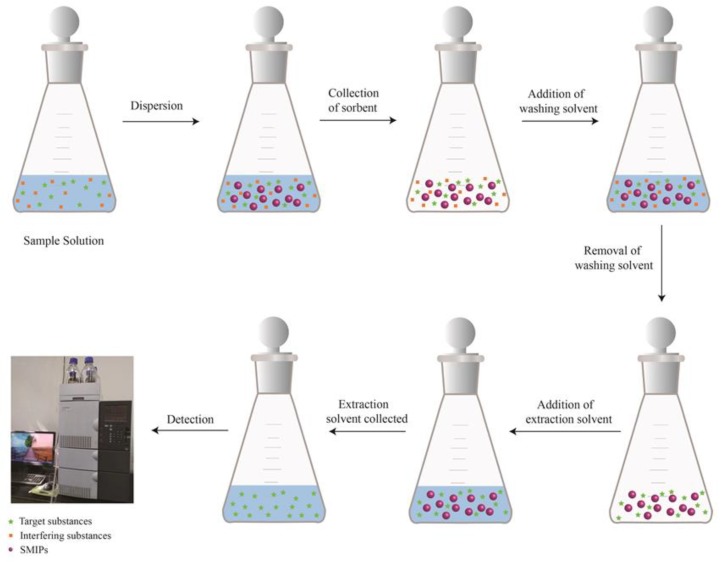
Graphical abstract of dispersive solid phase extraction (DSPE) method.

The DSPE procedure for extraction of GSF was as followed: 50 mg of SMIPs was dispersed in 5 mL spiked surface water sample in a 10 mL reaction flask. The reaction flask was shaken for 30 min at 150 revolutions per minute. After 30 min the supernatant was removed. After this washing solvent was added and allowed to disperse for 30 min. After 30 min, the solvent was removed and sorbent material remained in the flask. Further for extraction, 1 mL of desorption solvent was added in reaction flask and was shaken for 15 min. After 15 min, the supernatant was centrifuged, filtered, transferred into 1 mL vial, dried completely under nitrogen stream, reconstituted with 1 mL methanol and 10 µL of this solution was injected into HPLC for analysis. Figure 1 shows the procedure for selective extraction of GSF from surface water samples.

## 3. Results and Discussion

### 3.1. Removal of GSF from Surface Water

**Figure 2:**
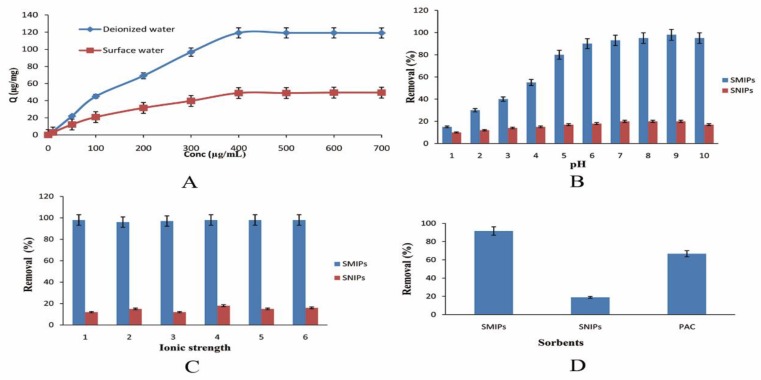
Removal of GSF. (**A**) Removal efficiency in surface and deionized water; (**B**) effect of pH on removal efficiency; (**C**) effect of ionization on removal efficiency; (**D**) comparison of different sorbent materials.

The practicality of applying SMIPs for the removal of GSF from surface water was measured by matching the adsorption isotherm in surface water and deionized water samples. The adsorption amount is a measure of removal efficiency. The adsorption amount of SMIPs in surface water sample was 76.4 µg/mg, while it was 119.1 µg/mg in deionized water (Figure 2A). The decline in the SMIPs adsorption efficiency for GSF in surface water may be ascribed to the differences in pH or higher amount of ions. Therefore, it was desired to determine the effect of pH and ionic content on the removal efficiency of SMIPs.

#### 3.1.1. Effect of pH

The removal efficiency of the drug largely depends upon pH value of water samples. Therefore, we determined the removal rate of GSF at different pH values. The results showed that removal rate of GSF range were maximum at pH value of 9 (Figure 2B). This may be due to the reason that the hydrophobic interaction and binding affinity of drug and specific sites at this pH range was maximum. Additionally, with an increase in pH, an increase in the adsorption of basic compounds to the methacrylic acid-type of polymers was resulted [35]. The removal efficiency of GSF decreased significantly with the decrease of pH when the pH was between 1–4. This phenomenon could be explained by the ionization of GSF. The GSF pKa value was >9 [16]. Ionization occurred for GSF under strong acidic condition. This resulted in the decrease of removal amount.

#### 3.1.2. Effect of Ionic Strength

We determined the effect of ionic strength on the removal efficiency of SMIPs. The results revealed that an increase or decrease of ionic strength did not significantly affect the removal rate of GSF (Figure 2C). In this ionic strength range (0–6%), the impact of the salting-out effect to GSF was too weak to exert any change in the removal of GSF on the SMIPs. Thus, the ionic strength of surface water samples was not supposed to exert any significant effect on the removal of GSF by SMIPs and SNIPs.

#### 3.1.3. Comparison of Removal Efficiency of SMIPs, SNIPs and Activated Carbon

The removal rate of GSF-SMIPs was compared with that of SNIPs, and PAC for the treatment of GSF. Figure 2D shows that the SMIPs had higher removal rate than SNIPs and PAC. The removal efficiency of SMIPs for GSF was higher (91.6%) than by PAC (67.7%). The removal efficiency of GSF did not increase by increasing the amount of PAC. The results demonstrated that SMIPs had a better removal efficiency and selectivity for GSF compared to PAC. Activated carbon removal has been used to remove pharmaceutical pollutants from water [36]. Moreover, the PAC was difficult to regenerate, and tended to saturate at lower loadings [37]. These favorable results made the SMIPs as a better choice for removal of GSF from contaminated waters.

### 3.2. Optimization of the DSPE Procedure

#### 3.2.1. Amount of SMIPs

**Figure 3:**
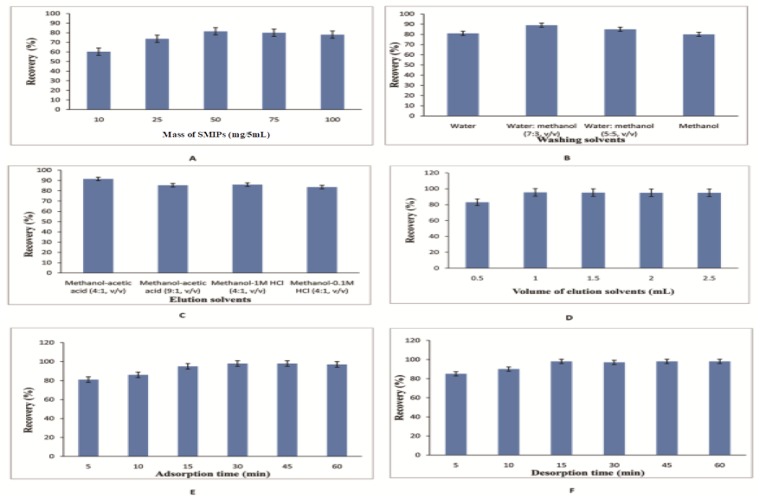
Optimization of DSPE procedure (**A**) Amount of sorbent; (**B**) washing solvents; (**C**) elution solvents; (**D**) volume of elution solvents; (**E**) time for adsorption; (**F**) time for desorption.

The amount of sorbent matrix had a major effect on the recoveries of target drugs from complex matrix. Therefore, the amounts of SMIPs were investigated in the range of 10–100 mg/5 mL. The results (Figure 3A) explained that maximum recovery achieved by using 50 mg/5 mL of SMIPs as sorbents. The recoveries of GSF were not increased by increasing the amount of SMIPs after 50 mg/5 mL. Therefore, 50 mg/5 mL were chosen as the final sorbent amount.

#### 3.2.2. Type and Volume of Washing Solvent

Different washing solvents were used to remove the interference substances and to obtain good recoveries. In this experiment, the washing solvents, including water, water: methanol (7:3, *v*/*v*), water: methanol (5:5, *v*/*v*) and methanol, were optimized. The volume of each washing solvent used in each washing step was 2 mL and maximum recoveries were as achieved (Figure 3B) by using water: methanol (7:3, *v*/*v*).

#### 3.2.3. Type and Volume of Elution Solvent

The effect of elution solvents on desorption of GSF from SMIPs was investigated. We used methanol as an elution solvent because the GSF is good soluble in methanol. An amount of acid was also optimized in desorption solvent. Subsequently, different types of elution solvents were applied such as methanol-acetic acid (4:1, *v*/*v*), methanol-acetic acid (9:1, *v*/*v*), methanol-1M HCl (4:1, *v*/*v*), methanol-0.1M HCl (4:1, *v*/*v*). The maximum recovery was achieved by using methanol-acetic acid (4:1, *v*/*v*) (Figure 3C). In the next step, we optimized the volume of elution solvent. Figure 3D shows a 1 mL volume gave maximum recovery of 95.4%.

#### 3.2.4. Adsorption and Desorption Time

The adsorption and desorption time have also been optimized for the efficient extraction of the GSF from samples. So it was studied by changing the vortex time from 5 to 60 min. The recoveries (Figure 3E) increased with the increase of adsorption time until it reached the equilibrium time. Finally, 30 min was chosen as the adsorption time. In this study, desorption time was also considered by increasing the vortex time from 5 to 60 min. The result (Figure 3F) shows that within 15 min the maximum recovery was achieved.

### 3.3. Selectivity Characteristics of SMIPs

**Figure 4:**
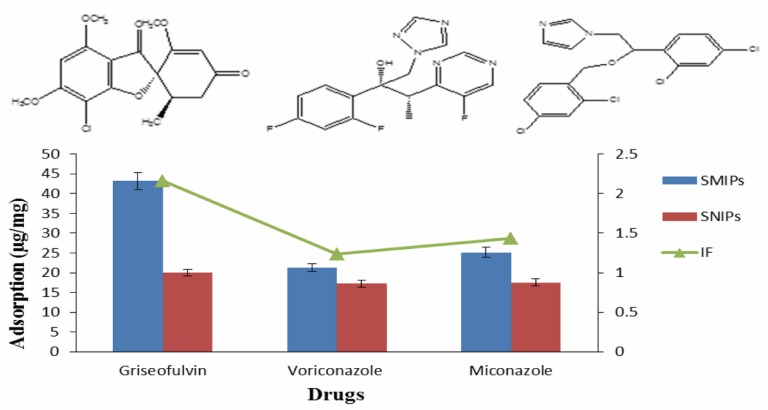
Selectivity of surface molecularly imprinted polymers (SMIPs) and surface non imprinted polymers (SNIPs).

We evaluated the selectivity of GSF SMIPs with voriconazole and miconazole in aqueous samples. As our main interest was the water remediation, so we determined the selectivity in aqueous phase. The imprint factor for GSF was 2.2 and for voriconazole was 1.2 and for miconazole was 1.4 (Figure 4). The SMIPs demonstrated lower recognition capability for therapeutic analogues. These results suggested the presence of specific sites on the surface of silica particles for GSF. These selective binding sites enhanced the adsorption of analyte from complex samples and decreased the binding of interfering substances [38].

### 3.4. Method Validation

The specificity of HPLC coupled to DSPE was evaluated with GSF standard solution, deionized and spiked surface water samples. The linear calibration curves of GSF in spiked surface water samples were obtained from the concentration range of 0.1–100 µg/mL with a linear calibration curve value (R^2^ = 0.9973). The limit of detection and limit of quantification were 0.01 µg/mL (signal to noise ratio (S/N), S/N = 3) and 0.03 µg/mL (S/N = 10). A good separation of GSF indicated that this method had a good specificity to detect GSF in surface water samples. Method accuracy and precision were established at three concentration levels of samples (0.1, 10, 50 µg/mL). The intra-day precision was evaluated by three repeated experiments of each spiked sample. Similarly, the inter-day precision was examined by recording the recoveries from spiked samples on three consecutive days. The relative standard deviation RSD- of intraday and inter-day precisions were less than 4.3% (Table 1).

### 3.5. Sample Application

**Figure 5:**
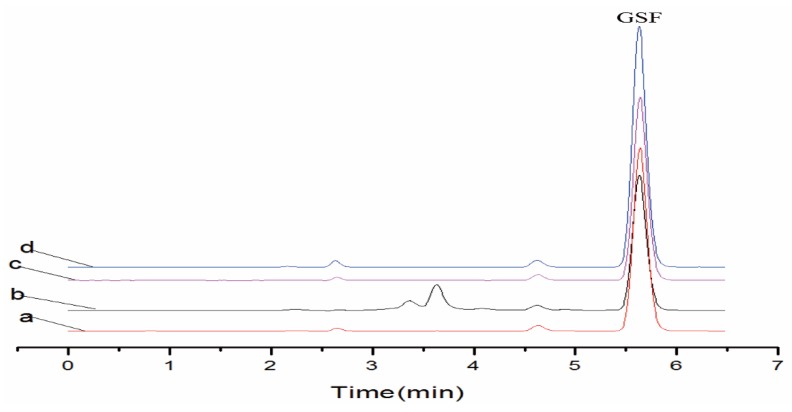
Recovery chromatograms of drug standard, surface and deionized water samples. (**a**) Drug standard 10 µg/mL; (**b**) spiked surface water sample by using SNIPs after DSPE; (**c**) spiked surface water sample by using SMIPs after DSPE; (**d**) spiked deionized water sample by using SMIPs after DSPE.

The water samples, spiked surface water samples and spiked deionized water samples were subjected to extraction by using DSPE method. The DSPE had good recoveries (91.6–98.8%) at the three concentration levels (Table 1). Figure 5 explains the chromatograms of standard drug sample, spiked surface water sample by using SNIPs after DSPE, spiked surface water samples by using SMIPs after DSPE and deionized water samples by using SMIPs after DSPE. The results of chromatogram (Figure 5) showed that after DSPE application there was a decrease of the amount of interference substances, and high enrichment of targets and good recoveries achieved by using SMIPs as sorbent material. So, a simple, selective, cost effective DSPE method can be used to extract, enrich, remove interface substances and detect the GSF from complex surface water samples.

### 3.6. Reusability of Sorbent

The SMIPs were employed to estimate the reusability and stability of SMIP-DSPE in aqueous samples. The result showed (Figure S1 that the SMIPs can be used ten times for binding/removing GSF in water samples. After ten cycles, the recoveries of GSF were all ≥73.1%. It indicated that SMIPs had a good reusability for water treatment and can be used several times without any significant performance loss.

### 3.7. Comparison with Previous Methods

The SMIP-DSPE method matched in extraction effectiveness and sensitivity with already reported methods [33,39,40,41]. The results (Table 2) validated that the developed SMIP-DSPE method has better recoveries as compared with previously reported methods. The method was fully validated according to the guidelines and presented a linear, specific, selective, reproducible and efficient method for detection and monitoring of GSF in water samples. In the DSPE method, less amount of sorbent and desorption solvent was used. Moreover extraction efficiency improved, extraction time reduced and a fast and reliable analytical method was established. The reusability of SMIPs in water samples was 10 times. Therefore, this DSPE extraction process is fast, selective, accurate, specific, simple, and reproducible.

## 4. Conclusions

In this work, GSF-SMIPs were successfully applied for the pre-concentration, extraction and removal of GSF from environmental water samples. SMIPs had better removal efficiency as compared to the activated carbon and can be used several times which demonstrated its clear advantage over other adsorbent materials. Under optimized conditions, a fast and sensitive method for the determination of GSF was established by coupling with HPLC. This method also offers better kinetic profile, performances and recoveries compared with previous established methods. The method has been applied for analysis of GSF in real water samples. The results suggest that SMIPs are superior and selective adsorbent material for the separation and detection of GSF in environmental water samples. This work provides a new method for the separation, monitoring and detection and removal of pharmaceutical pollutants from water samples.

## Figures and Tables

**Table 1:** Recovery and precision of griseofulvin (GSF) in deionized and surface water samples.

Sample	Spiked Drug Conc. (µg/mL)	Recoveries (%) *n* = 3	Precision (RSD %, *n* = 3)	
			Intra-Day	Inter-Day
Deionized Water	0.0	ND	0.0	0.0
	0.1	96.9	0.3	0.7
	10	98.8	0.2	0.8
	50	93.9	0.06	0.4
Surface Water	0.0	ND	0.0	0.0
	0.1	95.5	0.8	2.1
	10	98.6	1.5	3.5
	50	91.6	1.8	4.3

ND = not detected.

**Table 2:** Comparison with previous methods.

Method	Sample	Sample Volume Used	Precision	Limit of Detection (ng/mL)	Recoveries (%)	References
Gas Liquid Chromatography, Liquid-Liquid extraction GLC, LLE	Human Plasma	1	NA	6	97–107	39
LC-MS/MS, SPE	Human Plasma	0.5	7.5%	20 (LLOQ)	87.36	40
HPLC-Fluorescence, LLE	Rat Plasma	0.1	3.0–7.5%	1	99.20	41
HPLC-UV, SMISPE	Rat Plasma	0.5	0.9–4.5%	20	97.7	33
HPLC-UV, SMIP-DSPE	Surface Water Sample	5	0.2–4.3%	10	98.8	Current work

NA = not available; LLOQ = lowest limit of quantification.

## Supplementary Materials

The following are available online at https://www.mdpi.com/1660-4601/17/1/134/s1, Figure S1: Reusability of SMIPs.

## Abbreviations

DSPE	Dispersive solid phase extraction
GSF	Griseofulvin
IF	Imprinting factor
MIPs	Molecularly imprinted polymers
PAC	Powder activated carbon
SMIPs	Surface molecularly imprinted polymers
SNIPs	Surface non-molecularly imprinted polymers
SPE	Solid phase extraction
