# Supplementary figures and images for: Development of Surface Molecularly Imprinted Polymers as Dispersive Solid Phase Extraction Coupled with HPLC Method for the Removal and Detection of Griseofulvin in Surface Water
Source: Int J Environ Res Public Health. 2019 Dec 24;17(1):134. doi: 10.3390/ijerph17010134 (PMC6981569; doi:10.3390/ijerph17010134)

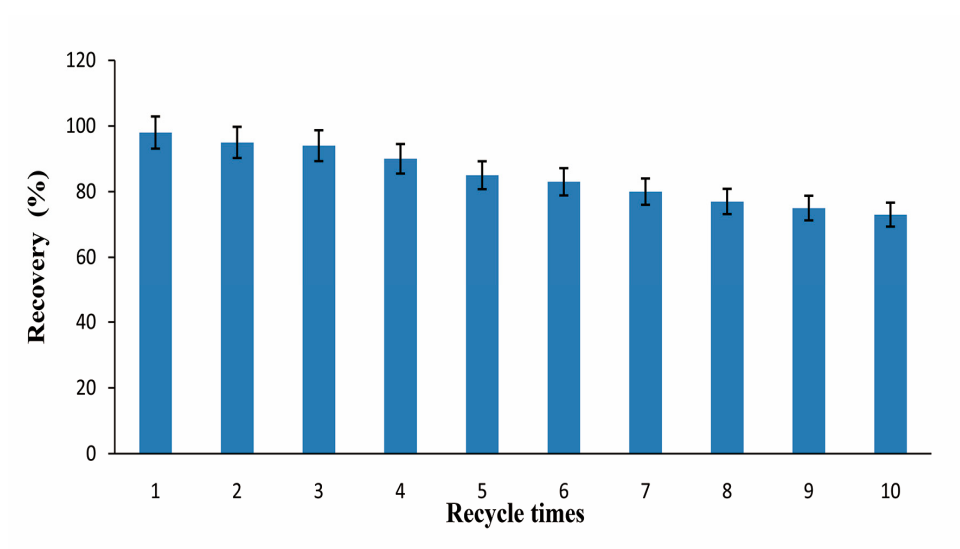

**Figure S1.** Reusability of SMIPs.

Supplement: Supplementary file 1 [file ijerph-17-00134-s001.pdf]
